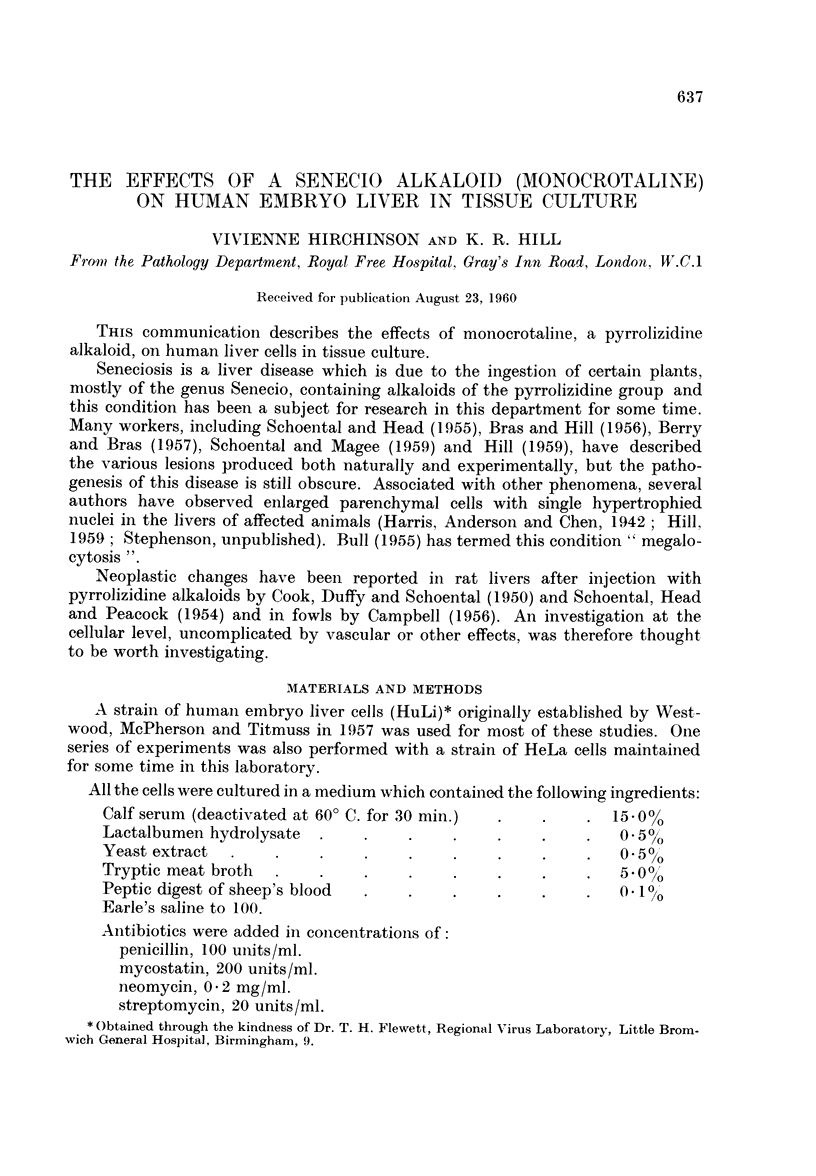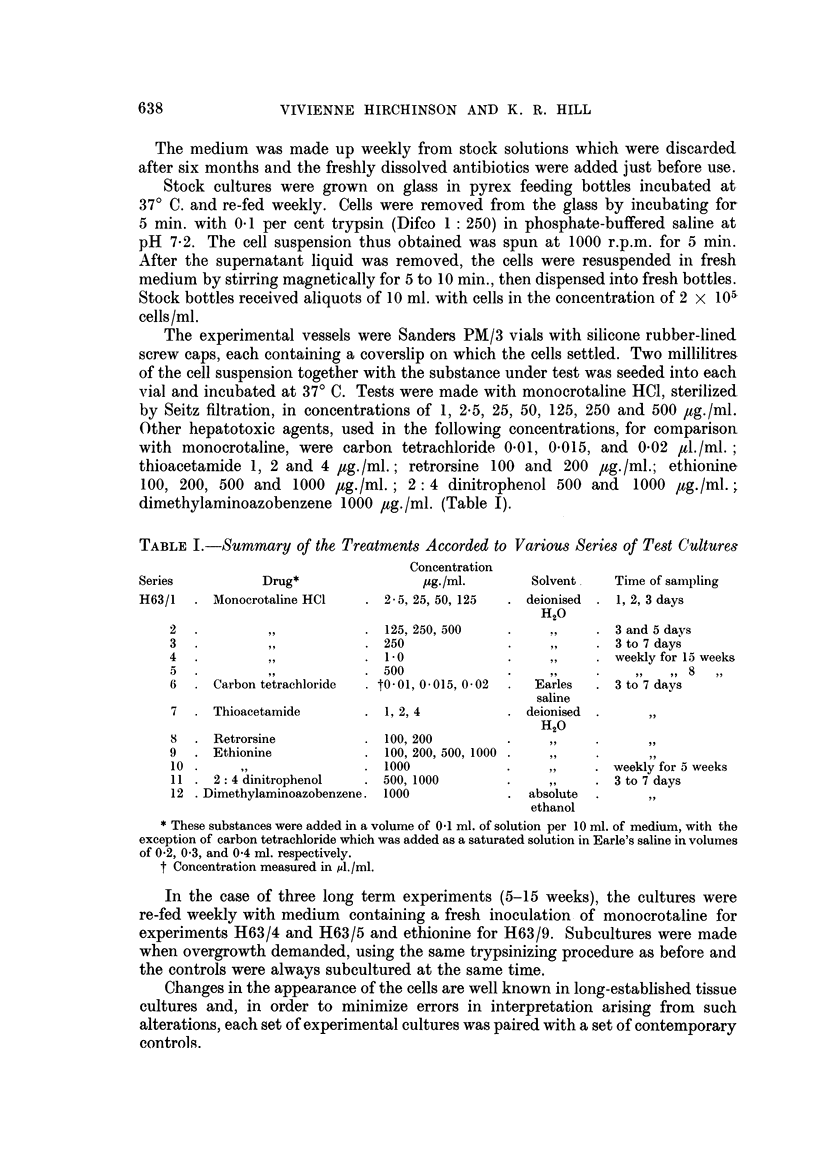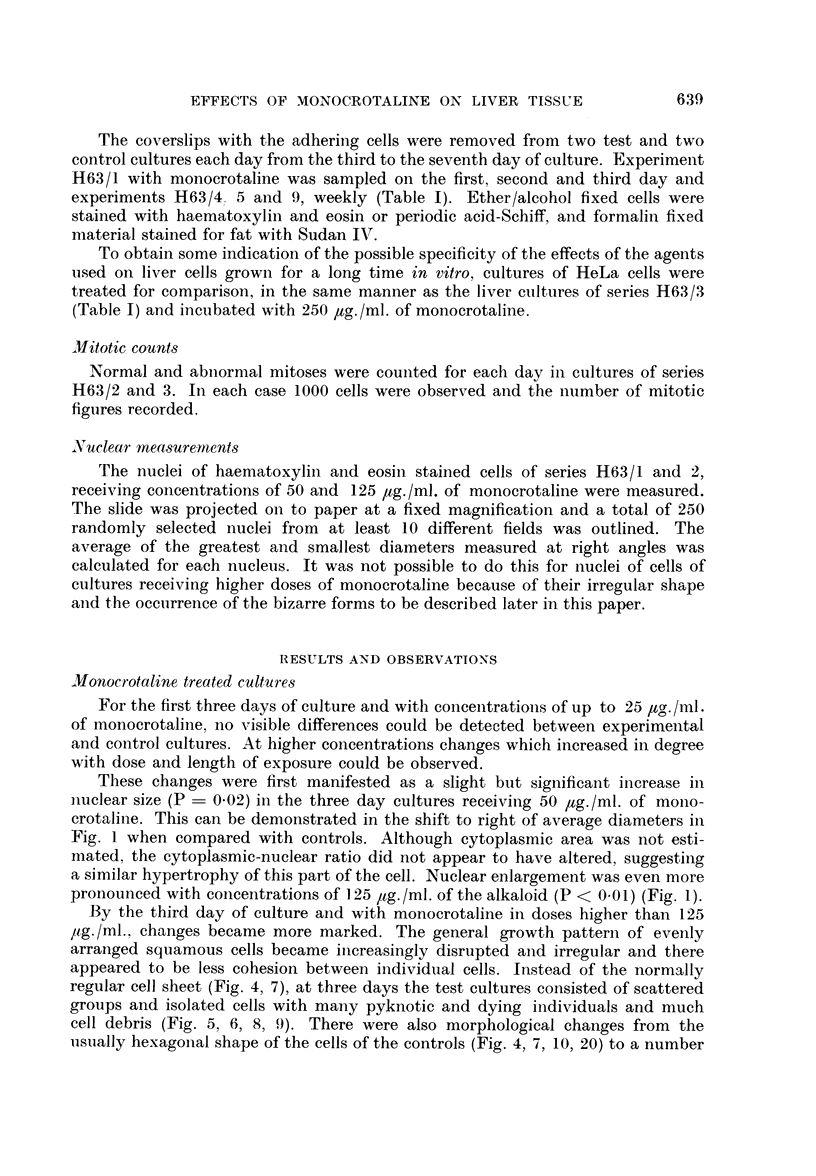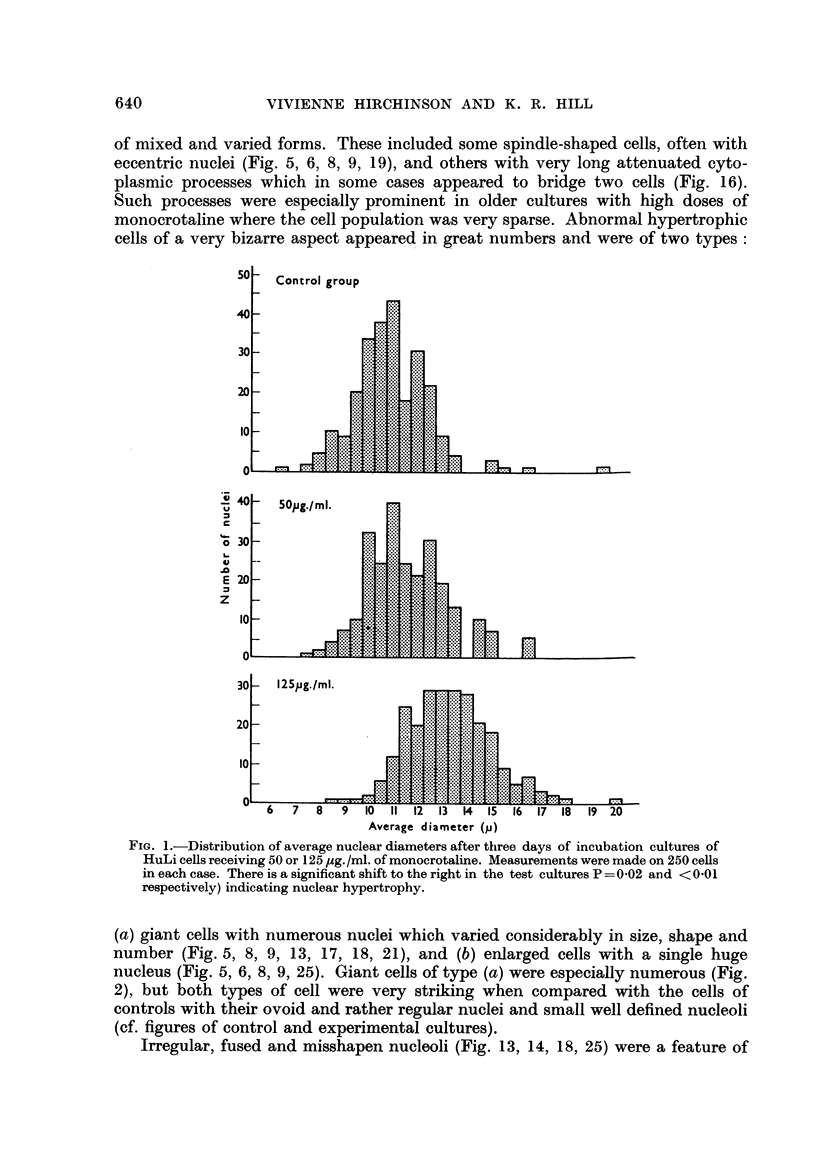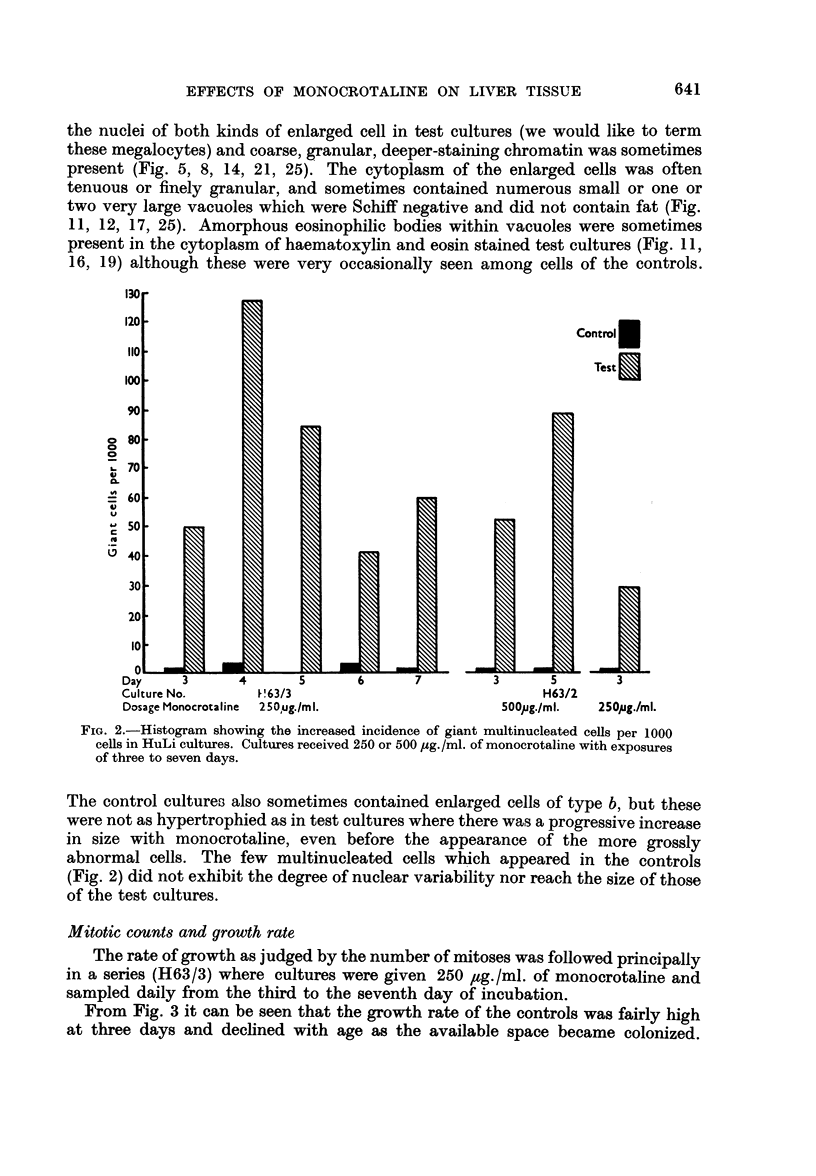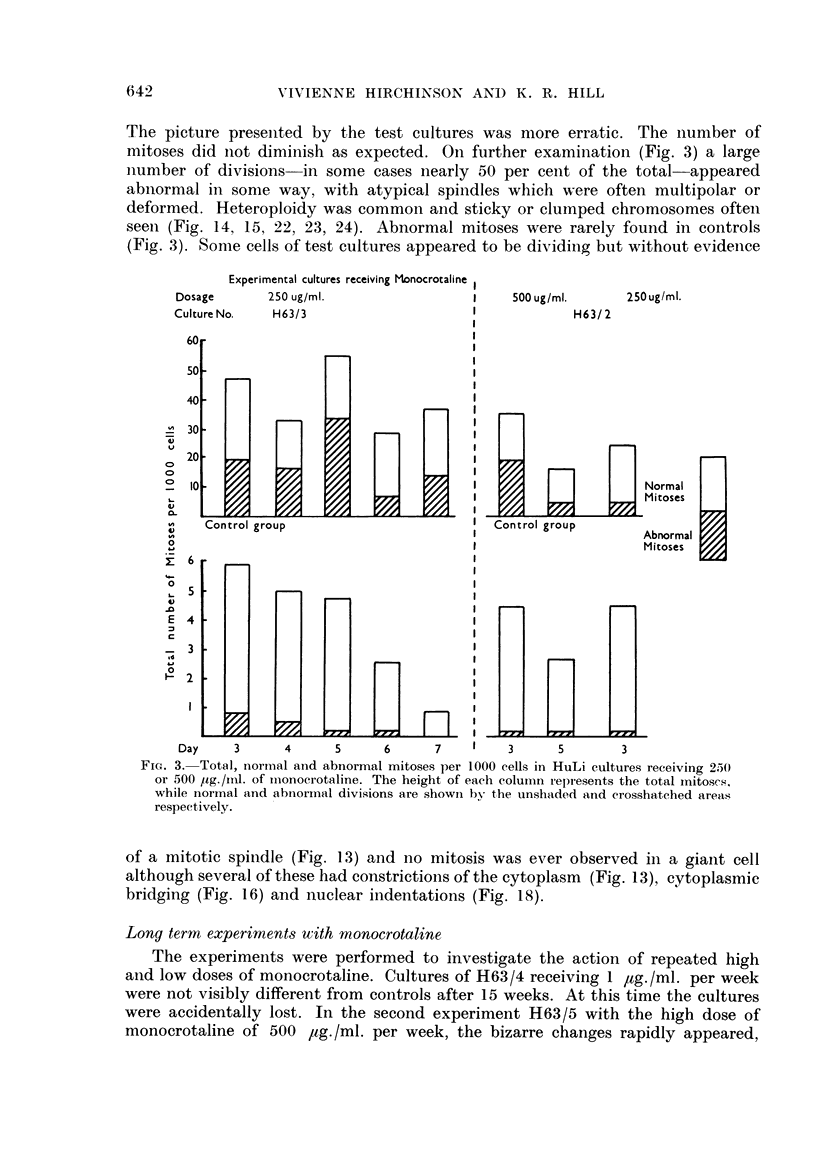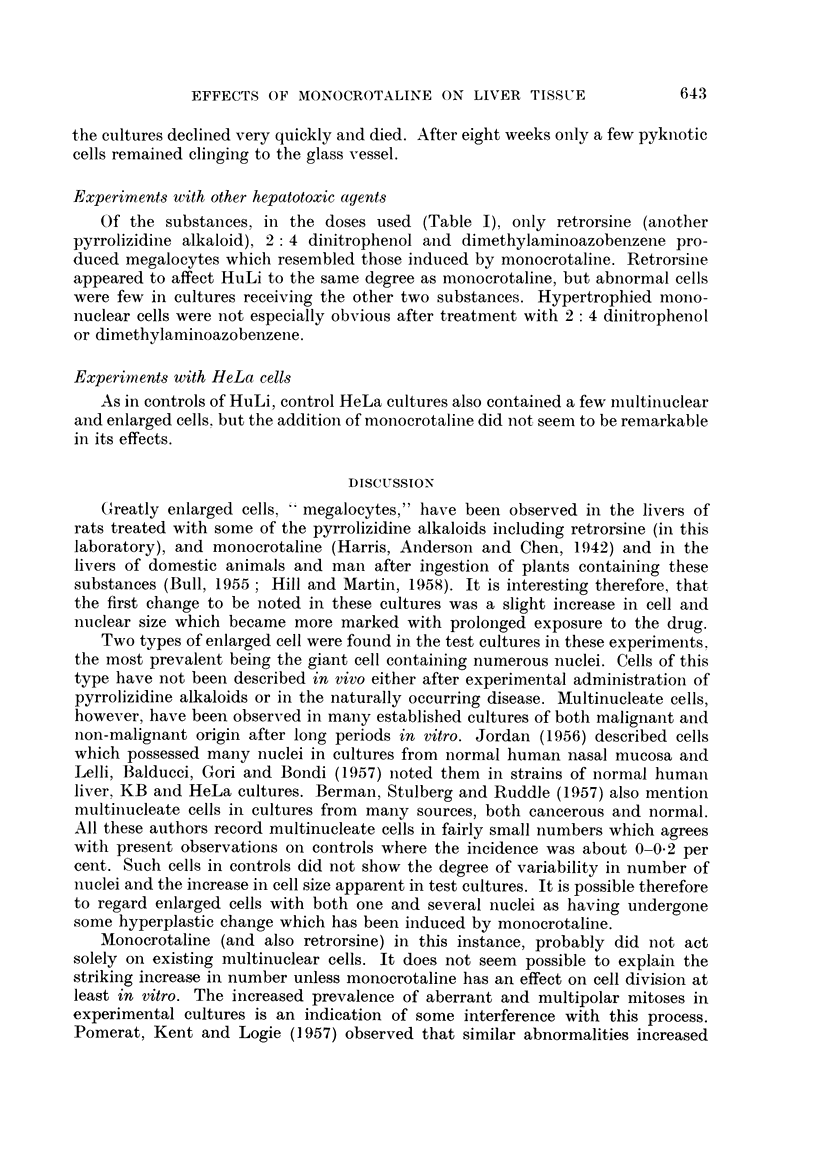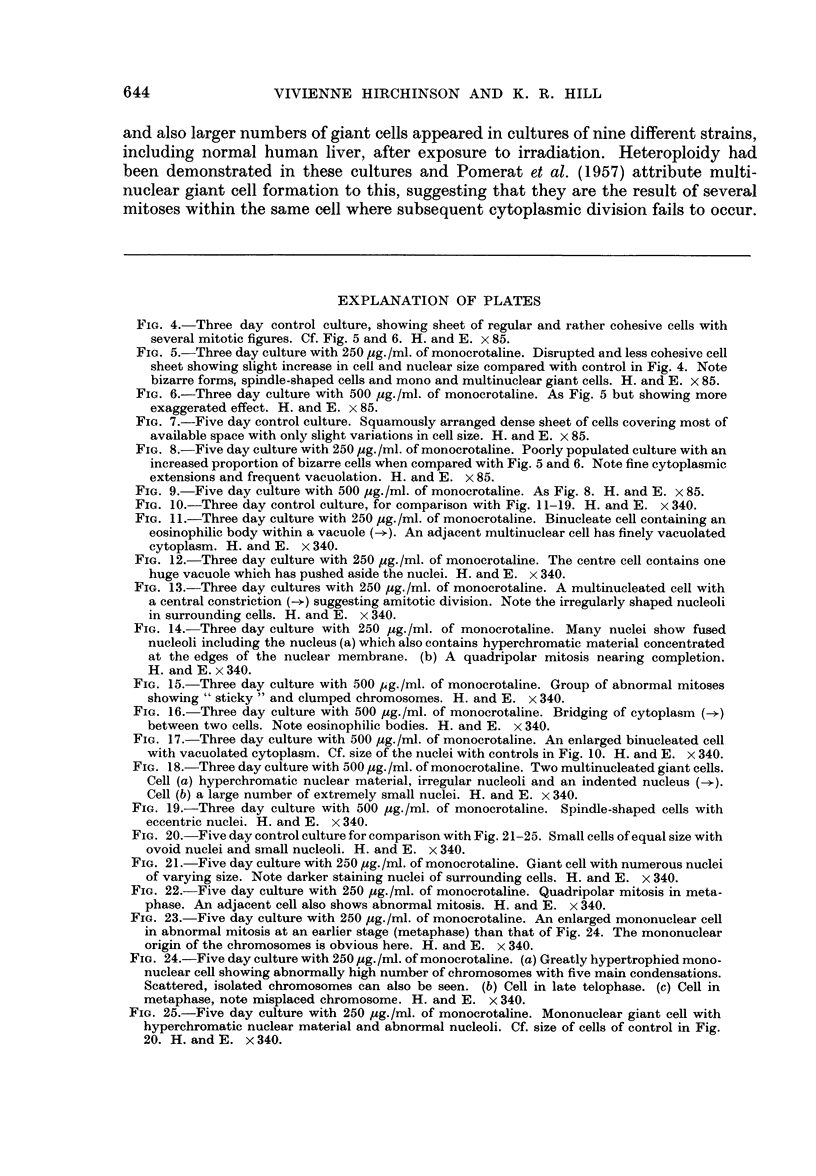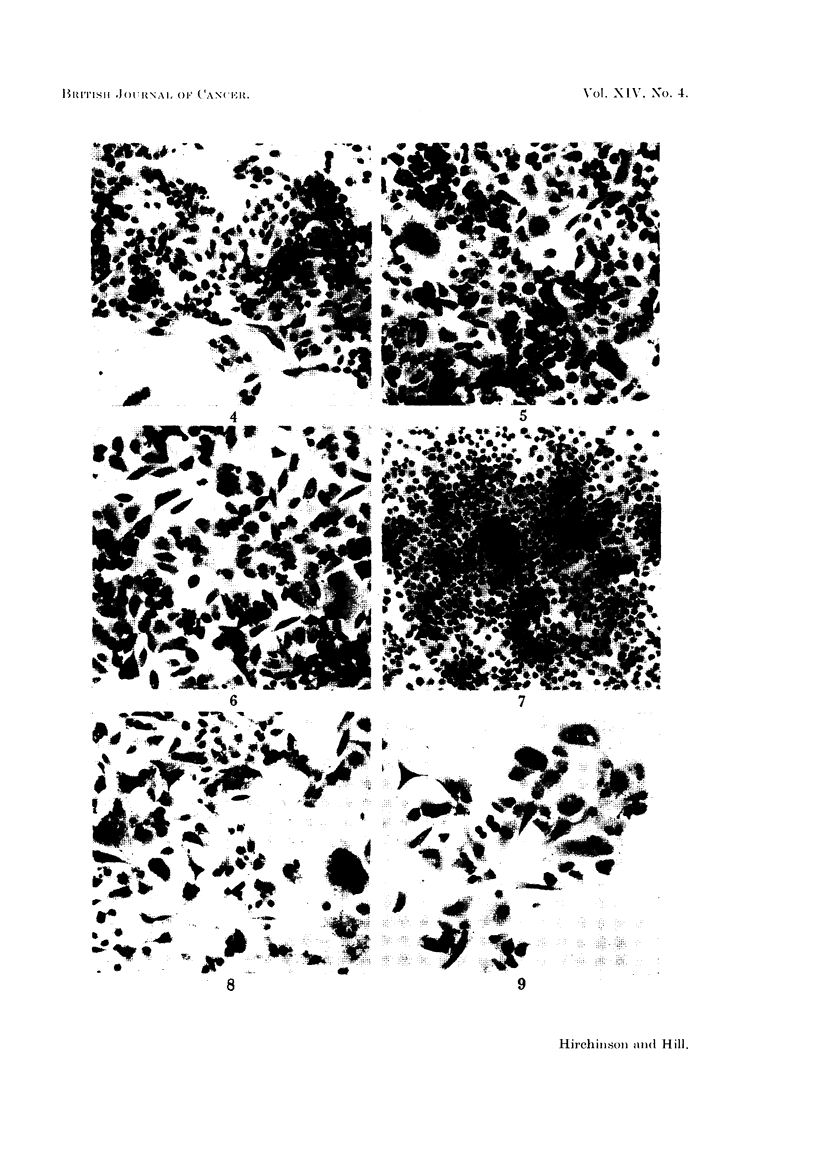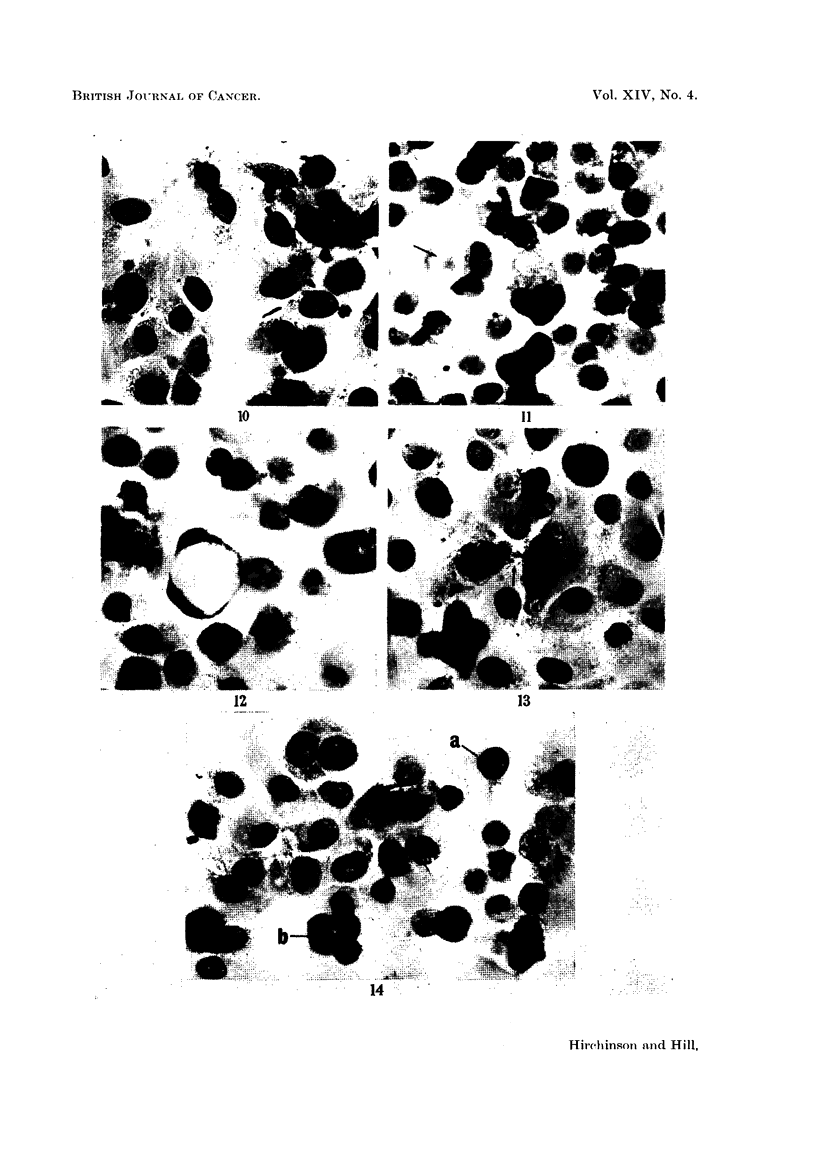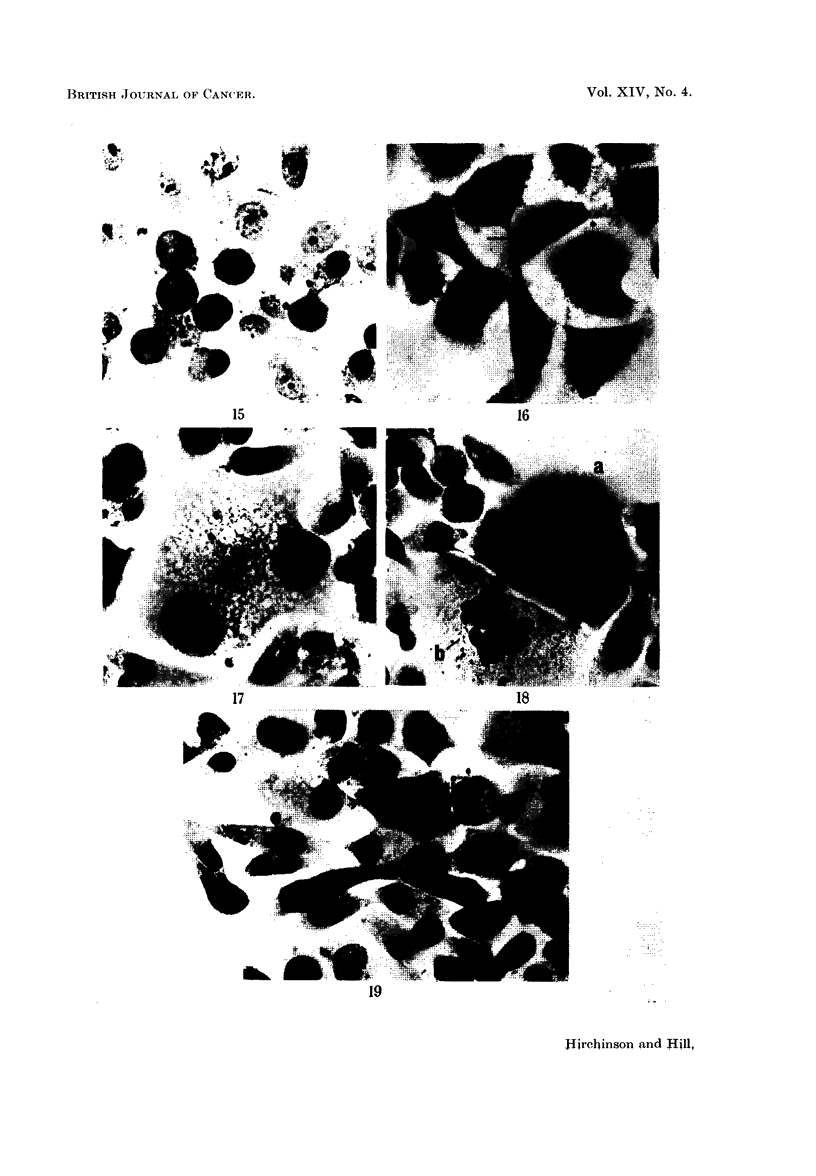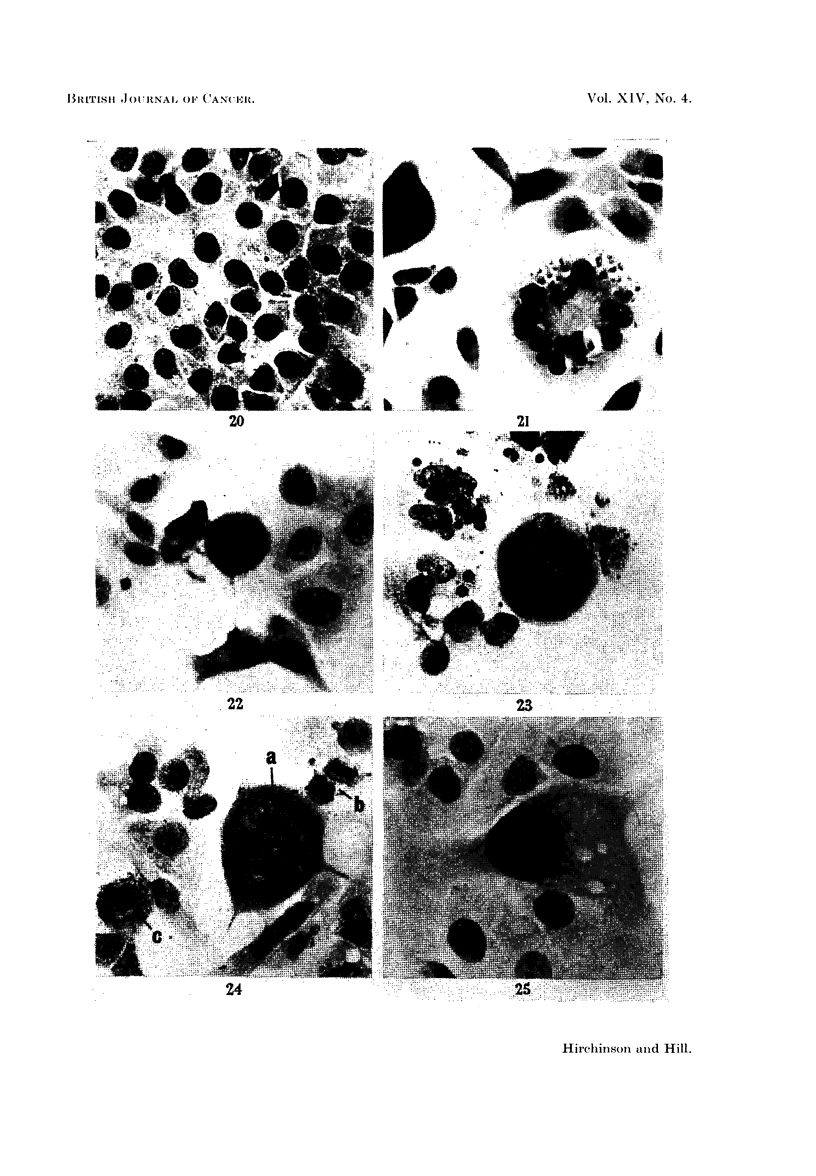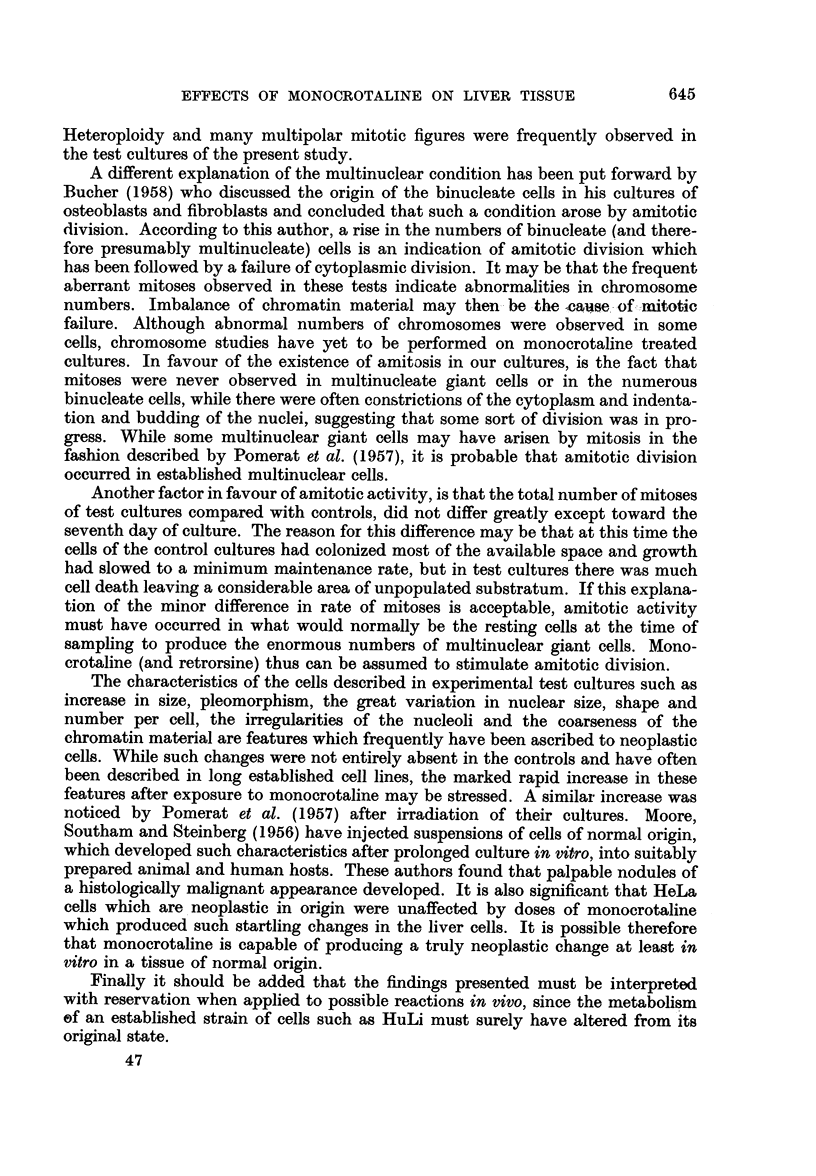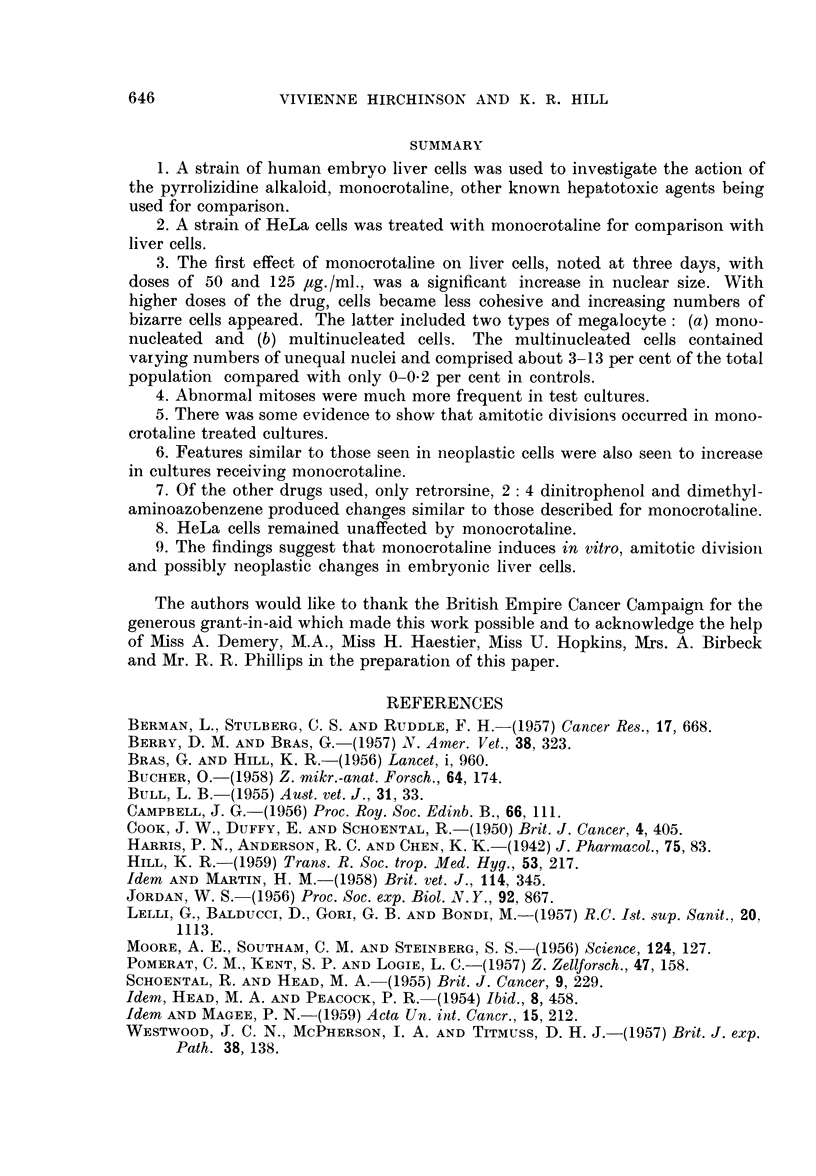# The Effects of a Senecio Alkaloid (Monocrotaline) on Human Embryo Liver in Tissue Culture

**DOI:** 10.1038/bjc.1960.70

**Published:** 1960-12

**Authors:** Vivienne Hirchinson, K. R. Hill

## Abstract

**Images:**


### THE EFFECTS OF A SENECIO ALKALOID (MONOCROTALINE) ON HUMAN EMBRYO LIVER IN TISSUE CULTURE

VIVIENNE HIRCHINSON AND K. R. HILL

*From the Pathology Department, Royal Free Hospital, Gray's Inn Road, London, W.C.1*

Received for publication August 23, 1960

THIS communication describes the effects of monocrotaline, a pyrrolizidine alkaloid, on human liver cells in tissue culture.

Seneciosis is a liver disease which is due to the ingestion of certain plants, mostly of the genus Senecio, containing alkaloids of the pyrrolizidine group and this condition has been a subject for research in this department for some time. Many workers, including Schoental and Head (1955), Bras and Hill (1956), Berry and Bras (1957), Schoental and Magee (1959) and Hill (1959), have described the various lesions produced both naturally and experimentally, but the pathogenesis of this disease is still obscure. Associated with other phenomena, several authors have observed enlarged parenchymal cells with single hypertrophied nuclei in the livers of affected animals (Harris, Anderson and Chen, 1942 ; Hill, 1959 ; Stephenson, unpublished). Bull (1955) has termed this condition " megalocytosis ".

Neoplastic changes have been reported in rat livers after injection with pyrrolizidine alkaloids by Cook, Duffy and Schoental (1950) and Schoental, Head and Peacock (1954) and in fowls by Campbell (1956). An investigation at the cellular level, uncomplicated by vascular or other effects, was therefore thought to be worth investigating.

### MATERIALS AND METHODS

A strain of human embryo liver cells (HuLi)* originally established by Westwood, McPherson and Titmuss in 1957 was used for most of these studies. One series of experiments was also performed with a strain of HeLa cells maintained for some time in this laboratory.

All the cells were cultured in a medium which contained the following ingredients:

| | |
|---|---|
| Calf serum (deactivated at 60° C. for 30 min.) | 15·0% |
| Lactalbumen hydrolysate | 0·5% |
| Yeast extract | 0·5% |
| Tryptic meat broth | 5·0% |
| Peptic digest of sheep's blood | 0·1% |
| Earle's saline to 100. | |

Antibiotics were added in concentrations of :
penicillin, 100 units/ml.
mycostatin, 200 units/ml.
neomycin, 0·2 mg/ml.
streptomycin, 20 units/ml.

* Obtained through the kindness of Dr. T. H. Flewett, Regional Virus Laboratory, Little Bromwich General Hospital, Birmingham, 9.

The medium was made up weekly from stock solutions which were discarded after six months and the freshly dissolved antibiotics were added just before use.

Stock cultures were grown on glass in pyrex feeding bottles incubated at 37° C. and re-fed weekly. Cells were removed from the glass by incubating for 5 min. with 0·1 per cent trypsin (Difco 1 : 250) in phosphate-buffered saline at pH 7·2. The cell suspension thus obtained was spun at 1000 r.p.m. for 5 min. After the supernatant liquid was removed, the cells were resuspended in fresh medium by stirring magnetically for 5 to 10 min., then dispensed into fresh bottles. Stock bottles received aliquots of 10 ml. with cells in the concentration of $2 \times 10^5$ cells/ml.

The experimental vessels were Sanders PM/3 vials with silicone rubber-lined screw caps, each containing a coverslip on which the cells settled. Two millilitres of the cell suspension together with the substance under test was seeded into each vial and incubated at 37° C. Tests were made with monocrotaline HCl, sterilized by Seitz filtration, in concentrations of 1, 2·5, 25, 50, 125, 250 and 500 $\mu$g./ml. Other hepatotoxic agents, used in the following concentrations, for comparison with monocrotaline, were carbon tetrachloride 0·01, 0·015, and 0·02 $\mu$l./ml.; thioacetamide 1, 2 and 4 $\mu$g./ml.; retrorsine 100 and 200 $\mu$g./ml.; ethionine 100, 200, 500 and 1000 $\mu$g./ml.; 2 : 4 dinitrophenol 500 and 1000 $\mu$g./ml.; dimethylaminoazobenzene 1000 $\mu$g./ml. (Table I).

TABLE I.—*Summary of the Treatments Accorded to Various Series of Test Cultures*

| Series | Drug* | Concentration $\mu$g./ml. | Solvent | Time of sampling |
|---|---|---|---|---|
| H63/1 | Monocrotaline HCl | 2·5, 25, 50, 125 | deionised $H_2O$ | 1, 2, 3 days |
| 2 | ,, | 125, 250, 500 | ,, | 3 and 5 days |
| 3 | ,, | 250 | ,, | 3 to 7 days |
| 4 | ,, | 1·0 | ,, | weekly for 15 weeks |
| 5 | ,, | 500 | ,, | ,, ,, 8 ,, |
| 6 | Carbon tetrachloride | †0·01, 0·015, 0·02 | Earles saline | 3 to 7 days |
| 7 | Thioacetamide | 1, 2, 4 | deionised $H_2O$ | ,, |
| 8 | Retrorsine | 100, 200 | ,, | ,, |
| 9 | Ethionine | 100, 200, 500, 1000 | ,, | ,, |
| 10 | ,, | 1000 | ,, | weekly for 5 weeks |
| 11 | 2 : 4 dinitrophenol | 500, 1000 | ,, | 3 to 7 days |
| 12 | Dimethylaminoazobenzene | 1000 | absolute ethanol | ,, |

* These substances were added in a volume of 0·1 ml. of solution per 10 ml. of medium, with the exception of carbon tetrachloride which was added as a saturated solution in Earle's saline in volumes of 0·2, 0·3, and 0·4 ml. respectively.

† Concentration measured in $\mu$l./ml.

In the case of three long term experiments (5–15 weeks), the cultures were re-fed weekly with medium containing a fresh inoculation of monocrotaline for experiments H63/4 and H63/5 and ethionine for H63/9. Subcultures were made when overgrowth demanded, using the same trypsinizing procedure as before and the controls were always subcultured at the same time.

Changes in the appearance of the cells are well known in long-established tissue cultures and, in order to minimize errors in interpretation arising from such alterations, each set of experimental cultures was paired with a set of contemporary controls.

The coverslips with the adhering cells were removed from two test and two control cultures each day from the third to the seventh day of culture. Experiment H63/1 with monocrotaline was sampled on the first, second and third day and experiments H63/4, 5 and 9, weekly (Table I). Ether/alcohol fixed cells were stained with haematoxylin and eosin or periodic acid-Schiff, and formalin fixed material stained for fat with Sudan IV.

To obtain some indication of the possible specificity of the effects of the agents used on liver cells grown for a long time *in vitro*, cultures of HeLa cells were treated for comparison, in the same manner as the liver cultures of series H63/3 (Table I) and incubated with 250 $\mu$g./ml. of monocrotaline.

*Mitotic counts*

Normal and abnormal mitoses were counted for each day in cultures of series H63/2 and 3. In each case 1000 cells were observed and the number of mitotic figures recorded.

*Nuclear measurements*

The nuclei of haematoxylin and eosin stained cells of series H63/1 and 2, receiving concentrations of 50 and 125 $\mu$g./ml. of monocrotaline were measured. The slide was projected on to paper at a fixed magnification and a total of 250 randomly selected nuclei from at least 10 different fields was outlined. The average of the greatest and smallest diameters measured at right angles was calculated for each nucleus. It was not possible to do this for nuclei of cells of cultures receiving higher doses of monocrotaline because of their irregular shape and the occurrence of the bizarre forms to be described later in this paper.

### RESULTS AND OBSERVATIONS

*Monocrotaline treated cultures*

For the first three days of culture and with concentrations of up to 25 $\mu$g./ml. of monocrotaline, no visible differences could be detected between experimental and control cultures. At higher concentrations changes which increased in degree with dose and length of exposure could be observed.

These changes were first manifested as a slight but significant increase in nuclear size ($P = 0{\cdot}02$) in the three day cultures receiving 50 $\mu$g./ml. of monocrotaline. This can be demonstrated in the shift to right of average diameters in Fig. 1 when compared with controls. Although cytoplasmic area was not estimated, the cytoplasmic-nuclear ratio did not appear to have altered, suggesting a similar hypertrophy of this part of the cell. Nuclear enlargement was even more pronounced with concentrations of 125 $\mu$g./ml. of the alkaloid ($P < 0{\cdot}01$) (Fig. 1).

By the third day of culture and with monocrotaline in doses higher than 125 $\mu$g./ml., changes became more marked. The general growth pattern of evenly arranged squamous cells became increasingly disrupted and irregular and there appeared to be less cohesion between individual cells. Instead of the normally regular cell sheet (Fig. 4, 7), at three days the test cultures consisted of scattered groups and isolated cells with many pyknotic and dying individuals and much cell debris (Fig. 5, 6, 8, 9). There were also morphological changes from the usually hexagonal shape of the cells of the controls (Fig. 4, 7, 10, 20) to a number of mixed and varied forms. These included some spindle-shaped cells, often with eccentric nuclei (Fig. 5, 6, 8, 9, 19), and others with very long attenuated cytoplasmic processes which in some cases appeared to bridge two cells (Fig. 16). Such processes were especially prominent in older cultures with high doses of monocrotaline where the cell population was very sparse. Abnormal hypertrophic cells of a very bizarre aspect appeared in great numbers and were of two types :

Fig. 1.—Distribution of average nuclear diameters after three days of incubation cultures of HuLi cells receiving 50 or 125 μg./ml. of monocrotaline. Measurements were made on 250 cells in each case. There is a significant shift to the right in the test cultures P = 0·02 and < 0·01 respectively) indicating nuclear hypertrophy.

(a) giant cells with numerous nuclei which varied considerably in size, shape and number (Fig. 5, 8, 9, 13, 17, 18, 21), and (b) enlarged cells with a single huge nucleus (Fig. 5, 6, 8, 9, 25). Giant cells of type (a) were especially numerous (Fig. 2), but both types of cell were very striking when compared with the cells of controls with their ovoid and rather regular nuclei and small well defined nucleoli (cf. figures of control and experimental cultures).

Irregular, fused and misshapen nucleoli (Fig. 13, 14, 18, 25) were a feature of the nuclei of both kinds of enlarged cell in test cultures (we would like to term these megalocytes) and coarse, granular, deeper-staining chromatin was sometimes present (Fig. 5, 8, 14, 21, 25). The cytoplasm of the enlarged cells was often tenuous or finely granular, and sometimes contained numerous small or one or two very large vacuoles which were Schiff negative and did not contain fat (Fig. 11, 12, 17, 25). Amorphous eosinophilic bodies within vacuoles were sometimes present in the cytoplasm of haematoxylin and eosin stained test cultures (Fig. 11, 16, 19) although these were very occasionally seen among cells of the controls.

FIG. 2.—Histogram showing the increased incidence of giant multinucleated cells per 1000 cells in HuLi cultures. Cultures received 250 or 500 µg./ml. of monocrotaline with exposures of three to seven days.

The control cultures also sometimes contained enlarged cells of type *b*, but these were not as hypertrophied as in test cultures where there was a progressive increase in size with monocrotaline, even before the appearance of the more grossly abnormal cells. The few multinucleated cells which appeared in the controls (Fig. 2) did not exhibit the degree of nuclear variability nor reach the size of those of the test cultures.

*Mitotic counts and growth rate*

The rate of growth as judged by the number of mitoses was followed principally in a series (H63/3) where cultures were given 250 µg./ml. of monocrotaline and sampled daily from the third to the seventh day of incubation.

From Fig. 3 it can be seen that the growth rate of the controls was fairly high at three days and declined with age as the available space became colonized.

The picture presented by the test cultures was more erratic. The number of mitoses did not diminish as expected. On further examination (Fig. 3) a large number of divisions—in some cases nearly 50 per cent of the total—appeared abnormal in some way, with atypical spindles which were often multipolar or deformed. Heteroploidy was common and sticky or clumped chromosomes often seen (Fig. 14, 15, 22, 23, 24). Abnormal mitoses were rarely found in controls (Fig. 3). Some cells of test cultures appeared to be dividing but without evidence

FIG. 3.—Total, normal and abnormal mitoses per 1000 cells in HuLi cultures receiving 250 or 500 μg./ml. of monocrotaline. The height of each column represents the total mitoses, while normal and abnormal divisions are shown by the unshaded and crosshatched areas respectively.

of a mitotic spindle (Fig. 13) and no mitosis was ever observed in a giant cell although several of these had constrictions of the cytoplasm (Fig. 13), cytoplasmic bridging (Fig. 16) and nuclear indentations (Fig. 18).

*Long term experiments with monocrotaline*

The experiments were performed to investigate the action of repeated high and low doses of monocrotaline. Cultures of H63/4 receiving 1 μg./ml. per week were not visibly different from controls after 15 weeks. At this time the cultures were accidentally lost. In the second experiment H63/5 with the high dose of monocrotaline of 500 μg./ml. per week, the bizarre changes rapidly appeared, the cultures declined very quickly and died. After eight weeks only a few pyknotic cells remained clinging to the glass vessel.

*Experiments with other hepatotoxic agents*

Of the substances, in the doses used (Table I), only retrorsine (another pyrrolizidine alkaloid), 2 : 4 dinitrophenol and dimethylaminoazobenzene produced megalocytes which resembled those induced by monocrotaline. Retrorsine appeared to affect HuLi to the same degree as monocrotaline, but abnormal cells were few in cultures receiving the other two substances. Hypertrophied mononuclear cells were not especially obvious after treatment with 2 : 4 dinitrophenol or dimethylaminoazobenzene.

*Experiments with HeLa cells*

As in controls of HuLi, control HeLa cultures also contained a few multinuclear and enlarged cells, but the addition of monocrotaline did not seem to be remarkable in its effects.

### DISCUSSION

Greatly enlarged cells, "megalocytes," have been observed in the livers of rats treated with some of the pyrrolizidine alkaloids including retrorsine (in this laboratory), and monocrotaline (Harris, Anderson and Chen, 1942) and in the livers of domestic animals and man after ingestion of plants containing these substances (Bull, 1955 ; Hill and Martin, 1958). It is interesting therefore, that the first change to be noted in these cultures was a slight increase in cell and nuclear size which became more marked with prolonged exposure to the drug.

Two types of enlarged cell were found in the test cultures in these experiments, the most prevalent being the giant cell containing numerous nuclei. Cells of this type have not been described *in vivo* either after experimental administration of pyrrolizidine alkaloids or in the naturally occurring disease. Multinucleate cells, however, have been observed in many established cultures of both malignant and non-malignant origin after long periods *in vitro*. Jordan (1956) described cells which possessed many nuclei in cultures from normal human nasal mucosa and Lelli, Balducci, Gori and Bondi (1957) noted them in strains of normal human liver, KB and HeLa cultures. Berman, Stulberg and Ruddle (1957) also mention multinucleate cells in cultures from many sources, both cancerous and normal. All these authors record multinucleate cells in fairly small numbers which agrees with present observations on controls where the incidence was about 0–0·2 per cent. Such cells in controls did not show the degree of variability in number of nuclei and the increase in cell size apparent in test cultures. It is possible therefore to regard enlarged cells with both one and several nuclei as having undergone some hyperplastic change which has been induced by monocrotaline.

Monocrotaline (and also retrorsine) in this instance, probably did not act solely on existing multinuclear cells. It does not seem possible to explain the striking increase in number unless monocrotaline has an effect on cell division at least *in vitro*. The increased prevalence of aberrant and multipolar mitoses in experimental cultures is an indication of some interference with this process. Pomerat, Kent and Logie (1957) observed that similar abnormalities increased and also larger numbers of giant cells appeared in cultures of nine different strains, including normal human liver, after exposure to irradiation. Heteroploidy had been demonstrated in these cultures and Pomerat *et al.* (1957) attribute multinuclear giant cell formation to this, suggesting that they are the result of several mitoses within the same cell where subsequent cytoplasmic division fails to occur.

---

### EXPLANATION OF PLATES

FIG. 4.—Three day control culture, showing sheet of regular and rather cohesive cells with several mitotic figures. Cf. Fig. 5 and 6. H. and E. ×85.

FIG. 5.—Three day culture with 250 μg./ml. of monocrotaline. Disrupted and less cohesive cell sheet showing slight increase in cell and nuclear size compared with control in Fig. 4. Note bizarre forms, spindle-shaped cells and mono and multinuclear giant cells. H. and E. ×85.

FIG. 6.—Three day culture with 500 μg./ml. of monocrotaline. As Fig. 5 but showing more exaggerated effect. H. and E. ×85.

FIG. 7.—Five day control culture. Squamously arranged dense sheet of cells covering most of available space with only slight variations in cell size. H. and E. ×85.

FIG. 8.—Five day culture with 250 μg./ml. of monocrotaline. Poorly populated culture with an increased proportion of bizarre cells when compared with Fig. 5 and 6. Note fine cytoplasmic extensions and frequent vacuolation. H. and E. ×85.

FIG. 9.—Five day culture with 500 μg./ml. of monocrotaline. As Fig. 8. H. and E. ×85.

FIG. 10.—Three day control culture, for comparison with Fig. 11–19. H. and E. ×340.

FIG. 11.—Three day culture with 250 μg./ml. of monocrotaline. Binucleate cell containing an eosinophilic body within a vacuole (→). An adjacent multinuclear cell has finely vacuolated cytoplasm. H. and E. ×340.

FIG. 12.—Three day culture with 250 μg./ml. of monocrotaline. The centre cell contains one huge vacuole which has pushed aside the nuclei. H. and E. ×340.

FIG. 13.—Three day cultures with 250 μg./ml. of monocrotaline. A multinucleated cell with a central constriction (→) suggesting amitotic division. Note the irregularly shaped nucleoli in surrounding cells. H. and E. ×340.

FIG. 14.—Three day culture with 250 μg./ml. of monocrotaline. Many nuclei show fused nucleoli including the nucleus (a) which also contains hyperchromatic material concentrated at the edges of the nuclear membrane. (b) A quadripolar mitosis nearing completion. H. and E. ×340.

FIG. 15.—Three day culture with 500 μg./ml. of monocrotaline. Group of abnormal mitoses showing " sticky " and clumped chromosomes. H. and E. ×340.

FIG. 16.—Three day culture with 500 μg./ml. of monocrotaline. Bridging of cytoplasm (→) between two cells. Note eosinophilic bodies. H. and E. ×340.

FIG. 17.—Three day culture with 500 μg./ml. of monocrotaline. An enlarged binucleated cell with vacuolated cytoplasm. Cf. size of the nuclei with controls in Fig. 10. H. and E. ×340.

FIG. 18.—Three day culture with 500 μg./ml. of monocrotaline. Two multinucleated giant cells. Cell (*a*) hyperchromatic nuclear material, irregular nucleoli and an indented nucleus (→). Cell (*b*) a large number of extremely small nuclei. H. and E. ×340.

FIG. 19.—Three day culture with 500 μg./ml. of monocrotaline. Spindle-shaped cells with eccentric nuclei. H. and E. ×340.

FIG. 20.—Five day control culture for comparison with Fig. 21–25. Small cells of equal size with ovoid nuclei and small nucleoli. H. and E. ×340.

FIG. 21.—Five day culture with 250 μg./ml. of monocrotaline. Giant cell with numerous nuclei of varying size. Note darker staining nuclei of surrounding cells. H. and E. ×340.

FIG. 22.—Five day culture with 250 μg./ml. of monocrotaline. Quadripolar mitosis in metaphase. An adjacent cell also shows abnormal mitosis. H. and E. ×340.

FIG. 23.—Five day culture with 250 μg./ml. of monocrotaline. An enlarged mononuclear cell in abnormal mitosis at an earlier stage (metaphase) than that of Fig. 24. The mononuclear origin of the chromosomes is obvious here. H. and E. ×340.

FIG. 24.—Five day culture with 250 μg./ml. of monocrotaline. (*a*) Greatly hypertrophied mononuclear cell showing abnormally high number of chromosomes with five main condensations. Scattered, isolated chromosomes can also be seen. (*b*) Cell in late telophase. (*c*) Cell in metaphase, note misplaced chromosome. H. and E. ×340.

FIG. 25.—Five day culture with 250 μg./ml. of monocrotaline. Mononuclear giant cell with hyperchromatic nuclear material and abnormal nucleoli. Cf. size of cells of control in Fig. 20. H. and E. ×340.

4

5

6

7

8

9

Hirchinson and Hill.

10

11

12

13

14

15

16

17

18

19

20

21

22

23

24

25

Heteroploidy and many multipolar mitotic figures were frequently observed in the test cultures of the present study.

A different explanation of the multinuclear condition has been put forward by Bucher (1958) who discussed the origin of the binucleate cells in his cultures of osteoblasts and fibroblasts and concluded that such a condition arose by amitotic division. According to this author, a rise in the numbers of binucleate (and therefore presumably multinucleate) cells is an indication of amitotic division which has been followed by a failure of cytoplasmic division. It may be that the frequent aberrant mitoses observed in these tests indicate abnormalities in chromosome numbers. Imbalance of chromatin material may then be the cause of mitotic failure. Although abnormal numbers of chromosomes were observed in some cells, chromosome studies have yet to be performed on monocrotaline treated cultures. In favour of the existence of amitosis in our cultures, is the fact that mitoses were never observed in multinucleate giant cells or in the numerous binucleate cells, while there were often constrictions of the cytoplasm and indentation and budding of the nuclei, suggesting that some sort of division was in progress. While some multinuclear giant cells may have arisen by mitosis in the fashion described by Pomerat *et al.* (1957), it is probable that amitotic division occurred in established multinuclear cells.

Another factor in favour of amitotic activity, is that the total number of mitoses of test cultures compared with controls, did not differ greatly except toward the seventh day of culture. The reason for this difference may be that at this time the cells of the control cultures had colonized most of the available space and growth had slowed to a minimum maintenance rate, but in test cultures there was much cell death leaving a considerable area of unpopulated substratum. If this explanation of the minor difference in rate of mitoses is acceptable, amitotic activity must have occurred in what would normally be the resting cells at the time of sampling to produce the enormous numbers of multinuclear giant cells. Monocrotaline (and retrorsine) thus can be assumed to stimulate amitotic division.

The characteristics of the cells described in experimental test cultures such as increase in size, pleomorphism, the great variation in nuclear size, shape and number per cell, the irregularities of the nucleoli and the coarseness of the chromatin material are features which frequently have been ascribed to neoplastic cells. While such changes were not entirely absent in the controls and have often been described in long established cell lines, the marked rapid increase in these features after exposure to monocrotaline may be stressed. A similar increase was noticed by Pomerat *et al.* (1957) after irradiation of their cultures. Moore, Southam and Steinberg (1956) have injected suspensions of cells of normal origin, which developed such characteristics after prolonged culture *in vitro*, into suitably prepared animal and human hosts. These authors found that palpable nodules of a histologically malignant appearance developed. It is also significant that HeLa cells which are neoplastic in origin were unaffected by doses of monocrotaline which produced such startling changes in the liver cells. It is possible therefore that monocrotaline is capable of producing a truly neoplastic change at least *in vitro* in a tissue of normal origin.

Finally it should be added that the findings presented must be interpreted with reservation when applied to possible reactions *in vivo*, since the metabolism of an established strain of cells such as HuLi must surely have altered from its original state.

SUMMARY

1. A strain of human embryo liver cells was used to investigate the action of the pyrrolizidine alkaloid, monocrotaline, other known hepatotoxic agents being used for comparison.

2. A strain of HeLa cells was treated with monocrotaline for comparison with liver cells.

3. The first effect of monocrotaline on liver cells, noted at three days, with doses of 50 and 125 μg./ml., was a significant increase in nuclear size. With higher doses of the drug, cells became less cohesive and increasing numbers of bizarre cells appeared. The latter included two types of megalocyte : (*a*) mononucleated and (*b*) multinucleated cells. The multinucleated cells contained varying numbers of unequal nuclei and comprised about 3–13 per cent of the total population compared with only 0–0·2 per cent in controls.

4. Abnormal mitoses were much more frequent in test cultures.

5. There was some evidence to show that amitotic divisions occurred in monocrotaline treated cultures.

6. Features similar to those seen in neoplastic cells were also seen to increase in cultures receiving monocrotaline.

7. Of the other drugs used, only retrorsine, 2 : 4 dinitrophenol and dimethylaminoazobenzene produced changes similar to those described for monocrotaline.

8. HeLa cells remained unaffected by monocrotaline.

9. The findings suggest that monocrotaline induces *in vitro*, amitotic division and possibly neoplastic changes in embryonic liver cells.

The authors would like to thank the British Empire Cancer Campaign for the generous grant-in-aid which made this work possible and to acknowledge the help of Miss A. Demery, M.A., Miss H. Haestier, Miss U. Hopkins, Mrs. A. Birbeck and Mr. R. R. Phillips in the preparation of this paper.

### REFERENCES

Berman, L., Stulberg, C. S. and Ruddle, F. H.—(1957) *Cancer Res.*, **17**, 668.
Berry, D. M. and Bras, G.—(1957) *N. Amer. Vet.*, **38**, 323.
Bras, G. and Hill, K. R.—(1956) *Lancet*, i, 960.
Bucher, O.—(1958) *Z. mikr.-anat. Forsch.*, **64**, 174.
Bull, L. B.—(1955) *Aust. vet. J.*, **31**, 33.
Campbell, J. G.—(1956) *Proc. Roy. Soc. Edinb.* B., **66**, 111.
Cook, J. W., Duffy, E. and Schoental, R.—(1950) *Brit. J. Cancer*, **4**, 405.
Harris, P. N., Anderson, R. C. and Chen, K. K.—(1942) *J. Pharmacol.*, **75**, 83.
Hill, K. R.—(1959) *Trans. R. Soc. trop. Med. Hyg.*, **53**, 217.
*Idem* and Martin, H. M.—(1958) *Brit. vet. J.*, **114**, 345.
Jordan, W. S.—(1956) *Proc. Soc. exp. Biol. N.Y.*, **92**, 867.
Lelli, G., Balducci, D., Gori, G. B. and Bondi, M.—(1957) *R.C. Ist. sup. Sanit.*, **20**, 1113.
Moore, A. E., Southam, C. M. and Steinberg, S. S.—(1956) *Science*, **124**, 127.
Pomerat, C. M., Kent, S. P. and Logie, L. C.—(1957) *Z. Zellforsch.*, **47**, 158.
Schoental, R. and Head, M. A.—(1955) *Brit. J. Cancer*, **9**, 229.
*Idem*, Head, M. A. and Peacock, P. R.—(1954) *Ibid.*, **8**, 458.
*Idem* and Magee, P. N.—(1959) *Acta Un. int. Cancr.*, **15**, 212.
Westwood, J. C. N., McPherson, I. A. and Titmuss, D. H. J.—(1957) *Brit. J. exp. Path.* **38**, 138.